# Influence of Cell Size and DNA Content on Growth Rate and Photosystem II Function in Cryptic Species of *Ditylum brightwellii*

**DOI:** 10.1371/journal.pone.0052916

**Published:** 2012-12-31

**Authors:** Susan C. Sharpe, Julie A. Koester, Martina Loebl, Amanda M. Cockshutt, Douglas A. Campbell, Andrew J. Irwin, Zoe V. Finkel

**Affiliations:** 1 Chemistry and Biochemistry, Mount Allison University, Sackville, New Brunswick, Canada; 2 School of Oceanography, University of Washington, Seattle, Washington, United States of America; 3 Center for Marine Environmental Sciences (MARUM), Bremen International Graduate School for Marine Sciences (GLOMAR), University of Bremen, Bremen, Germany; 4 Biology, Mount Allison University, Sackville, New Brunswick, Canada; 5 Mathematics and Computer Science, Mount Allison University, Sackville, New Brunswick, Canada; 6 Environmental Science Program, Mount Allison University, Sackville, New Brunswick, Canada; University of Connecticut, United States of America

## Abstract

DNA content and cell volume have both been hypothesized as controls on metabolic rate and other physiological traits. We use cultures of two cryptic species of *Ditylum brightwellii* (West) Grunow with an approximately two-fold difference in genome size and a small and large culture of each clone obtained by isolating small and large cells to compare the physiological consequences of size changes due to differences in DNA content and reduction in cell size following many generations of asexual reproduction. We quantified the growth rate, the functional absorption cross-section of photosystem II (PSII), susceptibility of PSII to photoinactivation, PSII repair capacity, and PSII reaction center proteins D1 (PsbA) and D2 (PsbD) for each culture at a range of irradiances. The species with the smaller genome has a higher growth rate and, when acclimated to growth-limiting irradiance, has higher PSII repair rate capacity, PSII functional optical absorption cross-section, and PsbA per unit protein, relative to the species with the larger genome. By contrast, cell division rates vary little within clonal cultures of the same species despite significant differences in average cell volume. Given the similarity in cell division rates within species, larger cells within species have a higher demand for biosynthetic reductant. As a consequence, larger cells within species have higher numbers of PSII per unit protein (PsbA), since PSII photochemically generates the reductant to support biosynthesis. These results suggest that DNA content, as opposed to cell volume, has a key role in setting the differences in maximum growth rate across diatom species of different size while PSII content and related photophysiological traits are influenced by both growth rate and cell size.

## Introduction

An organism’s size has a strong influence on its eco-physiological traits from maximum growth rate to grazing susceptibility [1], [2], [3]. Metabolic rate (*M*, often expressed as power in Watts or as mol O or C organism^−1^ time^−1^) scales with organism size (*S*, often carbon mass, dry weight, or cell volume for unicellular organisms) with a size-scaling exponent *b*:
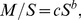
(1)where *c* can be a group-specific constant or include the temperature dependence of metabolic rates [2], [4], [5]. In phytoplankton, cell size ranges over 9 orders of magnitude [6], and has been shown to influence: light absorption efficiency [7], [8], susceptibility to photoinhibition [9], [10], nutrient diffusion and uptake kinetics [11], [12], and metabolic rates, including respiration [13], [14], [15], photosynthesis [8], [16] and growth [17], [18], [19], [20]. In addition, phytoplankton cell size influences food web structure and the sinking rate of organic matter from the ocean surface and therefore the biogeochemical cycling of elements such as carbon [21], [22].

The cause and value of the exponent associated with the size scaling of metabolism is a source of active debate [23], [24], [25], [26], [27], [28]. One hypothesis claims that the –1/4 size scaling of biomass normalized metabolic rates is universal and caused is by fundamental biophysical scaling constraints associated with metabolic networks applying to all organisms [23], [29], [30], [31]. It has been proposed that there is curvature in the logarithmic relationship between organism size and metabolic rate [26] and changes in the size-scaling exponent across broad taxonomic groups of different size [26], [32]. Alternatively the correlation between organism size and metabolic rate is hypothesized to be due to control by DNA content (C-value, the haploid genome size) and the volume of the nucleus [33], [34]. Definitive tests of these hypotheses using data from the literature are complicated by differences in experimental protocols and experimental or field conditions and phylogenetic and life-history structure in the data [24], [35], [36], [37].

Phylogenetic structure within datasets has been associated with variability in reported metabolic size-scaling exponents in a variety of higher taxonomic groups [24], [37], [38], [39]. In phytoplankton, compilations of metabolic rate and cell volume indicate that the size scaling of metabolic rate is often less than the proposed universal size-scaling exponent of –1/4 [1], [19], [32]. Different phylogenetic groups of organisms can have very different metabolic rates per unit biomass. For example, diatoms have on average mass-normalized metabolic rates that are two to three times that of dinoflagellates of the same size, and several fold higher than the Cyanophyceae picoplankton genera *Prochlorococcus* and *Synechococcus* [40]. Even within classes, phylogenetic bias can contribute to variability in estimates of the size scaling of metabolism [41]. Large differences in maximum and basal metabolic rates for different taxonomic groups that encompass different size ranges ensure that estimated metabolic size-scaling exponents will vary depending on the combination of species from different taxonomic groups included in the analysis.

Sub-optimal growth conditions, irradiance, temperature, and nutrient concentrations, can alter the size scaling of metabolism if the acquisition of resources limits metabolism and is size-dependent [5], [36], [42], [43]. Due to physical constraints larger cells will absorb fewer photons per unit of pigment than physiologically equivalent smaller cells with the same shape and intracellular pigment composition and concentration [44], [45]. As a consequence larger phytoplankton cells tend to have lower intracellular chlorophyll-*a* concentrations than smaller cells [7]. Larger cells are also less likely to become photoinhibited under high photon flux densities and UV exposure because they tend to have lower intracellular pigment concentrations and intercept fewer damaging photons per unit pigment than smaller cells [9], [10], [46]. Consequently pigment concentrations required to optimize growth will vary with the light regime and cell size, and the size scaling of growth and photosynthesis will vary with irradiance [8], [36].

Diatoms offer a useful experimental system in which to study the relationship between cell size, DNA content, physiological traits, and metabolic rate with a minimum of environmental and phylogenetic bias. Cell size decreases with each round of asexual division for most diatom species [47]. Over many rounds of asexual division the average width and volume of the diatom cells in culture decreases several fold, while the standard deviation increases [48], [49]. Eventually the diatom undergoes sexual reproduction, restoring cell size. Some species can also restore large cells to a population through a process of vegetative enlargement [50], [51]. The decrease in cell size with asexual division in diatoms permits the short-term establishment of cultures of clones with different average cell size and as a result the size scaling of metabolic rate and other physiological traits can be determined within a single species. Polyploidy has been reported in diatoms and may be an important mechanism fostering sympatric speciation, as in many plant and animal lineages [52], [53], [54]. Increases in DNA content (DNA per cell) are commonly associated with a corresponding positive linear increase in minimum cell volume and decreases in DNA content are associated with a corresponding linear decrease in cell volume [55]. Therefore speciation via changes in DNA content will alter cell volume and could have consequent impacts on metabolic rate and photophysiological traits [56]. Diatom species with known polyploids can be established in culture permitting an evaluation of the size scaling of metabolic rate and other physiological traits across closely related species differing in DNA content.

Using two putatively cryptic species of *Ditylum brightwellii* that share the same ITS1 rDNA, but which differ in average cell volume and DNA content [52] we determine how growth rate and photophysiological traits vary 1) with differences in cell volume due to asexual cell division over the life cycle within each cryptic species, and 2) with different DNA content across the closely related polyploids. This experiment allows us to discriminate between DNA content and cell volume control of the size scaling of metabolic rates.

## Materials and Methods

### Cultures, Growth Conditions and Growth Rate Determination

Two clones of *Ditylum brightwellii* (clone 17 and 19) were isolated from Puget Sound WA, USA and continued in culture from [52]. The clones were previously identified by their internal transcribed spacer 1 (ITS1) ribosomal DNA sequences and assigned to one of two populations, clone 17 from population 1 (P1) and clone 19 from population 2 (P2), but due to a ∼2-fold (1.93±0.74) average difference in genome size (see Fig. 4 in [52]) the two populations are putatively considered to be different cryptic species [52]. Clone 17 and 19 were grown in culture until a range of cell sizes was present, and then a small and a big cell were re-isolated by pipette and used to initiate new cultures. Subsequently small and big clonal cultures were maintained for both clone 17 and 19. The small and big cultures of *Ditylum brightwellii* P1 are referred to as P1S and P1B respectively, while P2S and P2B refer to small and big clonal cultures established from *Ditylum brightwellii* P2. The establishment of cultures of different average cell volume within P1 and P2 permits the evaluation of within species size scaling of traits, and differences in cell volume across P1 and P2 permits the evaluation of across (closely related) species size scaling of traits that may be due to differences in bulk DNA content. The average diameter of P1 and P2 used in this study (Table 1) are intermediate in diameter relative to values previously reported for the species [52], [57].

**Table 1:** Average (±2SE) size and photophysiological characteristics for a small and big isolate from two cryptic species, P1 and P2, of *Ditylum brightwellii* under different irradiances (I, µE m^2^s^−1^).

		P1		P2	
	I	small	big	small	big
Width (µm)		22.49±0.02	33.95±0.03	27.43±0.01	58.94±0.05
Length (µm)		50.39±0.08	53.94±0.08	55.09±0.08	80.49±0.09
Volume (µm^3^)		22,300±157	54,500±146	33,610±62	240,300±577
µ_max_ (day^−1^)		2.6±0.2	2.5±0.1	2.07±0.08	2.06±0.08
α_µ_(m^2^ mol photons^−1^)		0.23±0.04	0.24±0.04	0.21±0.02	0.18±0.02
σ_PSII_ (Å^2^ PSII^−1^)	37	261±19	255±11	232±36	189±15
R_PSII_ (s^−1^)	37	(1.94±0.34)⋅10^−4^	(1.77±0.03)⋅10^−4^	(1.38±0.41)⋅10^−4^	(1.36±0.41)⋅10^−4^
R_PSII_ (s^−1^)	287	(1.99±0.79)⋅10^−4^	(2.39±0.52)⋅10^−4^	(2.47±0.36)⋅10^−4^	(2.48±0.52)⋅10^−4^
σ_I_ (Å^2^ PSII^−1^)	37	(7.67±0.32)⋅10^−5^	(6.68±0.59)⋅10^−5^	(7.32±0.79)⋅10^−5^	(14.3±1.7)⋅10^−5^
σ_I_ (Å^2^ PSII^−1^)	287	(6.39±0.59)⋅10^−5^	(7.67±0.69)⋅10^−5^	(7.28±0.67)⋅10^−5^	(8.57±0.75)⋅10^−5^
PsbA(fmoles (µg protein)^–1^)	37	115.8±18.0	125.9±66.5	37.5±11.2	84.2±25.2
PsbA(fmoles (µg protein)^–1^)	287	70.3±16.7	87.5±6.5	25.7±9.6	54.2±62.4
PsbD(fmoles (µg protein)^–1^)	37	44.0±6.5	27.7±21.6	31.3±15.1	34.5±13.9
PsbD(fmoles (µg protein)^–1^)	287	24.4±9.1	14.4±10.2	28.6±4.1	17.2±16.7

The maximum growth rate µ_max_ and growth yield, *α*_µ_ are based on log_2_ estimates of growth rate as a function of irradiance.

*Ditylum brightwellii* was grown on f/2+ Si media [58] at 18°C, with a 12∶12 hour light:dark cycle using semi-continuous batch culture technique [59]. A batch culture for P1S, P1B, P2S, and P2B at each light treatment was used to establish the range of cell concentrations to maintain exponential growth. Cultures were kept optically thin, mainly <10^5^ cells per mL. All observations were made on cultures grown for at least five generations in mid-exponential phase. Growth rate was measured at five different irradiances: 37, 47, 92, 287 and 559 µmol photons m^−2^ s^−1^, in 3–6 different replicate bottles. Spherical scalar photosynthetic photon flux density was measured with a microspherical quantum sensor (Walz, Germany) connected to a Li-Cor Model LI-250 light meter (Lincoln, Nebraska, USA) or a QSL-2101 light meter from Biospherical Instruments Inc. (San Diego, CA, USA). Cells were counted daily near the start of the light period using a Sedgewick Rafter (SPI supplies, West Chester, PA, USA) counting chamber. Two or three counts were averaged, typically with a coefficient of variation of less than 10%. The growth rate (μ, day^−1^) for each sample was obtained from a linear regression analysis of the increase in log_2_ cell density (over mid-exponential phase) over time, typically using five points over five days.

### Cell Width, Diameter and Cell Volume

Using ImageJ (http://rsbweb.nih.gov/ij/index.html) approximately 100 measurements of cell length and width (used as an estimate of cell diameter) were taken from digital images of cells in valve view, from one replicate, for each isolate, at each irradiance. Images were taken with a FlowCAM (Fluid Imaging Technologies, Yarmouth, ME, USA) with a 200 µm flow cell. The size of the FlowCAM images was calibrated by running 20 µm latex beads through the machine. The size of the beads was confirmed using a light microscope and micrometer. Cell volume was calculated from the linear dimensions, assuming the cells were cylindrical. P2B was somewhat rectangular in valve view, and therefore the cylindrical approximation may have slightly under-estimated cell volume.

### Photosynthetic Parameters

The cross-section for PSII photoinactivation (σ_I_, Å^2^ PSII^−1^) and an estimate of PSII repair rate capacity (R_PSII_, s^−1^) in response to a short-term increase in irradiance were estimated for mid-exponential phase cultures acclimated under 37 and 287 µmol photons m^−2^ s^−1^ for a minimum of 5 generations for each of P1S, P1B, P2S and P2B, in triplicate bottles. The functional absorption cross-section for PSII (σ_PSII_, Å^2^ PSII^−1^), an estimate of the target size of PSII [60] was measured using a Satlantic FIRe (Satlantic, Halifax, Canada) for the species grown under 37 µmol photons m^−2^ s^−1^ and dark-adapted for 10 minutes before measurement following [61]. To estimate PSII repair capacity (R_PSII_) and the susceptibility of PSII to photoinactivation (σ_I_) each culture bottle was split into two flasks and dark-adapted for 10 minutes, with one flask incubated with lincomycin to block protein synthesis (repair) [62] at the initiation of the light challenge experiment. Both flasks were then exposed to a short-term light challenge of 450 µmol photons m^−2^ s^−1^ of blue light for 90 minutes and sampled at 15, 30, 60 and 90 minutes to measure PSII fluorescence parameters (R_PSII_, σ_I_) using a Xenon–PAM (Walz, Effetrich, Germany) following the protocols described in [61] and [9]. PSII repair capacity is estimated from the difference in the maximum quantum yield for PSII photochemistry, between the control and lincomycin treatments, after correcting for any influence of sustained phases of non-photochemical quenching; see [61] for additional detail. The cross-section for photoinactivation per PSII (σ_I_) was estimated from the exponential decrease in maximum quantum yield from the lincomycin treatment versus the cumulative photon dose incident on the culture [9], [63].

### Quantitation of Photosystem II Reaction Center Proteins Using Immunoblotting

Key proteins of the PSII complex, D1 and D2, were estimated as described in [64] using PsbA and PsbD, respectively. Cells were harvested on binder-free glass fiber filters (25 mm diameter), which were immediately flash-frozen in liquid nitrogen and stored at −80°C for later protein analyses by quantitative immunoblotting of PsbA and PsbD. Total protein was extracted and determined by three thawing/sonicating rounds in denaturing extraction buffer (Lowry protein assay kit, Bio-Rad DC Protein Assay, Bio-Rad Laboratories, Hercules, CA., USA). 1–2 µg of total protein were loaded on 4–12% acrylamide precast NuPAGE gels (Invitrogen Corporation, Carlsbad, CA., USA). Along with the samples, protein standards for each target protein (Agrisera, Vännäs, Sweden) were loaded to establish a standard curve. Electrophoresis was run for 30–35 minutes at 200 V in MES-SDS Running Buffer (Invitrogen) and the proteins were transferred to a PVDF membrane (80 minutes at 30 V in the Invitrogen SureLock XCell). After membrane blocking in 2% ECL Advance blocking agent, primary antibody against PsbA (Agrisera, 1∶40,000–1∶25,000) and PsbD (AgriSera, 1∶20,000–1∶25,000) were applied, followed by an anti-rabbit secondary antibody coupled with horseradish peroxidase (Immunoreagents, 1∶40,000–1∶25,000). The membranes were developed by chemiluminescence using ECL Advance (Amersham Biosciences, Quebec, Canada) and a CCD imager (Kodak 4000 MMPro, Carestream or Bio-Rad VersaDoc). Target protein concentrations were determined by fitting the sample signal values to the protein standard curves, taking care that all sample signals fell within the range of the protein standard curve, and that no band signals were saturated.

### Model Fitting and Determination of Size-scaling Exponents and Rationale for Determining the Average DNA and Cell Volume of P2 Relative to P1

Growth-irradiance (µ-*I*) parameters were derived from a non-linear least squares fit of the data to
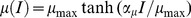
(2)
[65], where tanh is the hyperbolic tangent function, *µ*_max_ is the maximum growth rate (time^−1^) and the growth efficiency or growth yield, *α*_µ_ (m^2^ µmol photons^–1^), is the growth rate divided by growth irradiance as irradiance approaches zero.

The size-scaling exponent for growth rate as a function of cell volume was determined by ordinary least squares regression on the log_10_ transformed data, log *µ* = *a*+*b* log *V*, using a separate intercept, *a*, for each irradiance. First, we fit a separate metabolic size-scaling exponent, *b*, for each cryptic species to identify the size scaling effect due to variation in cell volume (*V*) associated with life cycle stage within P1 and P2. Second, to separate the size-scaling exponent of growth into two components due to the cell volume change determined by differences in DNA content and cell volume change due to rounds of asexual reproduction, we fit the following model: log *µ* = *a*+*b*_DNA_ log *V*_DNA_+*b*_A_ log *V*_A_, where we used two measures of cell volume: a volume effect across the two species with a 2-fold difference in DNA content, *V*_DNA_, equal to 1 for P1 and 2 for P2, according to their differences in cellular DNA content [52], and cell volume which varied within each species with life cycle stage, *V*_A_ = *V*/*V*_DNA_.

Previous work has documented that *Ditylum brightwellii* P2 has an average cellular DNA content 1.94±0.74 (1 SD) times that of P1 and an associated increase in cell size (for 22 clones examined in [52]). Koester et al. [52] measured the relative DNA content for the specific P1 and P2 strains used in this study; the average ratio of the DNA content of P2:P1 from this study is 1.93±0.37 (1 SD). Given the DNA ratio for P2:P1 is not significantly different from 2, for a wide variety of clones including the specific clones used in this study, it seems likely that there has been a genome duplication event and the DNA content in P2 is 2-times that of P1.

To estimate the size-scaling exponent associated with metabolic rates across species (P1 and P2), the average sizes of P1 and P2 should be used. Unfortunately the full size range of most diatom species, and P1 and P2, are not well characterized. Point estimates of cell volume from the clonal cultures in this study do not provide a reliable estimate of the average difference in cell volume across P1 and P2. For example, if our point estimate of cell volume in our P1B clones comes from the first quarter of their life cycle, the resulting estimate of mean cell volume will be larger than the true average cell volume, and, if our point estimate of cell volume in our P2S clones comes from the last quarter of their life cycle, the resulting estimate of mean cell volume will be smaller than the true average cell volume; this will bias our size-scaling exponent estimates. We have no information on the life cycle stage of our clonal cultures when our cell size estimates were made and we do not know quantitatively how cell volume varies with life cycle stage in our cultures; therefore we cannot use our point estimates of cell volume in P1 and P2 to estimate a reliable average cell volume ratio of P2 relative to P1. Alternatively, we use the difference in haploid DNA content (DNA per cell) to estimate the average cross-species (P2:P1) ratio in cell volume and mass. In unicellular eukaryotes average cell volume is proportional to DNA content to the power of 0.97 [55] and DNA content is proportional to cellular carbon content to the power 0.92 to 1.15 [41], [53], [66]. The 95% confidence interval for the size-scaling exponent for cellular carbon content of diatoms as a function of cell volume ranges from 0.79 to 0.97 [1], [67]. We therefore estimate the size-scaling (cell volume and mass) exponent associated with growth across P1 and P2 assuming there is 2-fold difference in DNA content, cell volume and cell carbon content across P1 and P2 in this study. Linear assumptions about the co-variation in cell volume, carbon content and DNA content are reasonable based on previous studies, but ideally co-incident measurements over the whole size range of multiple clonal lineages are required to confirm these assumptions as small changes in these relationships could significantly alter the estimated size-scaling exponents associated with growth and other metabolic rates.

## Results

### Cell Size within and Across Species of Ditylum Brightwellii

Cultures of *Ditylum brightwellii* P1 and P2 had significantly different cell widths, lengths and volumes, with narrow ranges (Table 1). The valve face was elliptical except in P2B where it was rectangular. Previous work indicates cell diameters in *D. brightwellii* can range from 5–110 µm [52], [57]. In this study cell widths in valve view (an estimate of diameter) range from 22.5 to 59 µm for P1 and P2 (Table 1), indicating that our clonal populations are intermediate in size for the species. Within species, P1B was 1.5 times wider than P1S, while P2B was 2.15 times wider than P2S. Cell widths also varied across the two species, with P2B being on average 1.7 times wider than P1B. There was no measurable change in the average cell width within a clone over a short time-scale of one to two weeks over which experimental measurements were made, but over the months that cultures were maintained cell width and volume decreased. For example, over two months, the average width of P2B decreased by 26% from 72±12 µm (2 SE) to 53±10 µm (2 SE), while the average width of P1S decreased by 18%, from 24.3±1.9 to 20.0±2.0 µm. Length varied over 2-fold within P1 and P2 over the cell division cycle and the average value in each population was measured in order to calculate volume. Cell volume varied significantly across and within the species: the volume of P2B was 10.8 times the volume of P1S. Volume varied within P2 by a factor of 7.2, and by a factor of 2.4 within P1 (Table 1). The species (P1) with the smaller genome (see Materials in Methods for details) has smaller minimum frustule width and average cell volume than P2.

### Growth Rate as a Function of Irradiance

**Figure 1:**
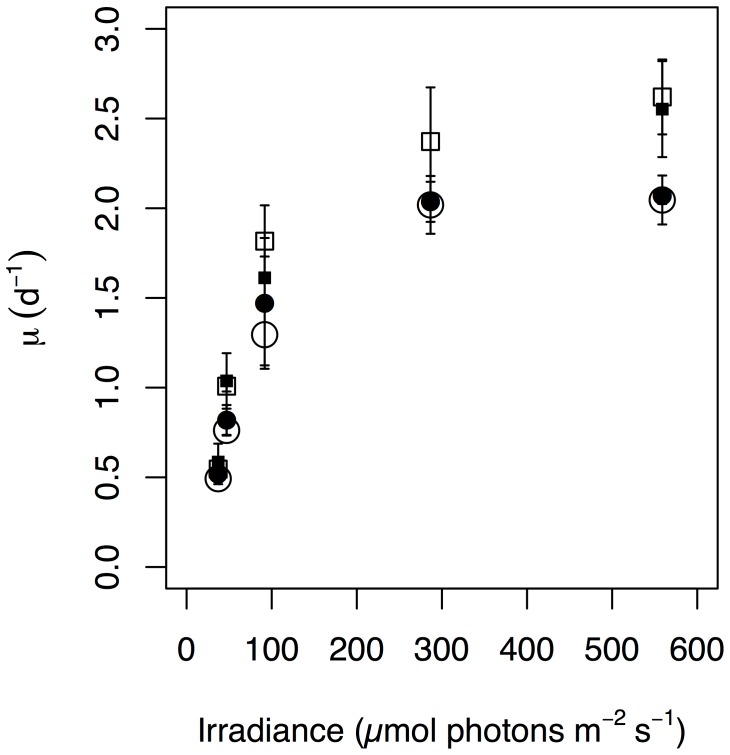
Steady-state acclimated log_2_ growth rate (μ±2SE, n≥3) as a function of irradiance for big (B, large open symbols) and small (S, small closed symbols) isolates from two species, P1 (squares) and P2 (circles) of *Ditylum brightwellii*. *Ditylum brightwellii* P2 has 1.93±0.37 times the DNA content of P1.

Under saturating growth irradiance *Ditylum brightwellii* achieves fast growth rates relative to many other diatoms given its size [19], [52], [57]. A large proportion of the cells in the P1S culture underwent sexual reproduction when grown at 287 µmol photons m^−2^ s^−1^, so no reliable asexual growth rate data was obtained for this light level. A minimum of 3 and maximum of 6 estimates of growth rate were made for each of the two species and two size-classes, at each irradiance (total n = 81). Growth rate showed a strong dependence on irradiance (Fig. 1), consistent with Equation 2. At all irradiances, P1 grew more quickly than P2. Maximum growth rate, µ_max_ (d^−1^), was significantly higher for P1 than P2 (Table 1). Within each species (P1S vs. P1B and P2S vs. P2B), there was very little variation in growth rate at a given irradiance, despite large and significant differences in cell volumes (Fig. 1). For example, despite having volumes that were 7 times larger, P2B had a growth rate not significantly different from the smaller P2S across all irradiances. It appears (Fig. 1) that there is an increase in the difference in growth rate between P1 and P2 with increasing irradiance, but the ratio of growth rates (P1/P2) and proportional differences in growth rates ((P1–P2)/P1) exhibit no clear pattern with irradiance. The growth rate efficiency, α_μ_ (m^2^ µmol photons^–1^), was higher in P1 relative to P2, and lowest in P2B, although differences within and across the species were not statistically significant (>2 SE).

### PSII Functional Absorption Cross-section, Repair Capacity, Cross-section for Photoinactivation, and PsbA and PsbD Content

**Figure 2:**
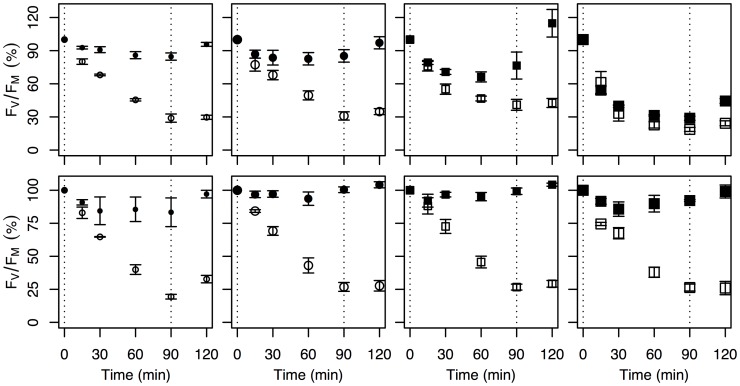
Light challenge experiments conducted on *Ditylum brightwellii* P1S, P1B, P2S, and P2B (left to right) acclimated to 37 (top panels) and 287 µmol photons m ^−**2**^ **s** ^−**1**^ **(bottom panels).** PSII repair capacity is estimated from the difference in *F*_V_/*F*_M_ between the control (filled symbol) and lincomycin (open symbol) treatments. The susceptibility to photoinactivation is estimated from the change in *F*_V_/*F*_M_ in the lincomycin treatment. Vertical dashed lines indicate the start (*t* = 0) and end (*t* = 90) of the high light challenge. *F*_V_/*F*_M_ measurements are relative to *F*_V_/*F*_M_ at t = 0.

Light challenge experiments and protein assays were performed on cultures grown in mid-exponential phase under growth limiting and saturating irradiance (I = 37 and 287 µmol photons m^−2^ s^−1^) to estimate the susceptibility of the PSII reaction center to photoinactivation (σ_I_) and its repair rate capacity (R_PSII_) as well as the pool size of the PSII reaction center proteins D1 (PsbA) and D2 (PsbD) under growth limiting and saturating irradiance. *Ditylum brightwellii* P1 had a larger PSII functional absorption cross-section (σ_PSII_) and higher PSII repair capacity (R_PSII_) relative to P2 when acclimated to the lower irradiance (Table 1, Fig. 2). When acclimated to growth-saturating irradiance and then exposed to a short-term increase in irradiance, R_PSII_ within P1 was comparable to values observed for the species acclimated to lower irradiance. In contrast R_PSII_ in P2 increased approximately 1.75-fold when acclimated to the higher versus lower irradiance. As a result there is little difference in R_PSII_ across or within species when acclimated to growth saturating irradiance. The cross-section for photoinactivation, σ_I_, an estimate of gross PSII inactivation (in the absence of repair) on the basis of cumulative photon dose, was similar for P1S, P1B, and P2S whether they were acclimated to growth limiting or saturating irradiances. P2B had the highest values of σ_I_, particularly when acclimated to low irradiance.

The concentration of PSII reaction center proteins D1 (PsbA) and D2 (PsbD) varied with irradiance and across species (Table 1). Most of the differences in PsbA and PsbD per µg protein were not significantly different across irradiance, across populations or across sizes within the populations. PsbA and PsbD per µg protein tended to be higher when acclimated to growth limiting versus growth saturating irradiances, both across and within species. In particular P1S has significantly higher values (>2 SE) of PsbA and PsbD per µg protein under 37 versus 287 µmol photons m^−2^ s^−1^. PsbA per µg protein tended to be higher in P1 relative to P2, independent of acclimation irradiance. PsbD per µg protein tended to be less dynamically variable with irradiance, and within or across species, compared to PsbA.

### The Size Scaling of Growth Rate as a Function of Cell Volume and DNA Content

Cell volume was estimated for 60 of the 81 growth rate measurements, resulting in 3 to 4 replicate estimates of growth rate with associated cell volume estimates for P1S, P1B, P2S, and P2B for each experimental irradiance, except that under 287 µmol photons m^−2^ s^−1^ P1S had no estimate of growth rate, and P1B had two growth rate replicates. At any given irradiance, there is little change in growth rate within a species, regardless of differences in cell width or volume, resulting in weak size scaling of growth within the species. The average size-scaling exponent for the cell volume normalized growth rate is *b* = –0.03±0.01 (2 SE) within each of the two species of *Ditylum brightwellii* (Table 2).

**Table 2:** Size-scaling exponents, *b*, from a linear regression of log growth rate µ = *a*+*b* log *V* using a different intercept, *a*, for each growth irradiance (not shown).

		Slope	Standard error	p-value	Root mean square error (d.f.)
**A**	Within P1	–0.024	0.014	0.09	0.036 (53)
	Within P2	–0.037	0.013	0.006	
	Average	–0.031	0.013		
	Multiple R^2^	0.98			
**B**	Within (*b*_A_)	–0.035	0.013	0.01	0.04 (53)
	Across (*b*_DNA_)	–0.23	0.032	<0.001	
	Multiple R^2^	0.98			

A) Different metabolic size-scaling exponents are calculated for P1 and P2. B) Separate size-scaling exponents are calculated within and across the two species of *Ditylum brightwellii*, assuming that P2 has 2-times the DNA content and volume of P1.

The size-scaling exponent is significantly larger when comparisons are made across the species. When we use a point estimate of cell volume from our cultures and compare P1S with P2S and P1B with P2B then the average *b* = –0.13±0.02 (2 SE). We recommend caution in interpreting this size-scaling exponent (*b* = –0.13±0.02) because it is unlikely that the cultures for each replicate and clone are in same stage in their life cycle; ideally the average (or minimum or maximum) size for each of the populations should be used in this calculation. If we instead assume P2 has an average cell volume 2-times that of P1 (based on their DNA content, see Materials and Methods for additional details and rationale), then the cross-species size-scaling exponent for growth as a function of cell volume is –0.23±0.03 (2 SE) (Table 2). If P2 is on average 1.93-times the cell volume of P1, based on differences in their DNA content, then the cross-species size-scaling (cell volume) exponent for growth is –0.24±0.034 (2 SE).

It was difficult to assess the possible impact of irradiance on the size scaling of growth rate because the growth rates of the small and big sized isolates were similar within the species; therefore, there were only two growth rates to compare at each irradiance.

## Discussion

There are several hypotheses for the theoretical origins for the size-scaling exponent associated with metabolic rates. One of the leading hypotheses proposes that metabolic rate is controlled by cell or organism volume due to biophysical scaling constraints associated with metabolic networks [23], [30], [31]. Alternatively it has been hypothesized that DNA content/nuclear volume sets minimum cell volume and metabolic rate [34]. To test these hypotheses with a minimum of environmental and phylogenetic bias we measured cell volume and a number of physiological traits, including growth rate, within and across two cryptic species of *Ditylum brightwellii* (P1 and P2) that differ in DNA content. We find that growth rate is highest in the cryptic species with lower DNA content (P1), regardless of photon flux density and cell size associated with life cycle stage. These results are consistent with previous work testing numerous *Ditylum brightwellii* clones from P1 and P2 [52]. Given a 2-fold difference in DNA content and cell mass across the two cryptic species, the size-scaling exponent associated with light-saturated growth rate is –0.23±0.03 (2SE). In contrast, within each of the cryptic species of *Ditylum brightwellii* (DNA content is constant) there is little variation in growth rate despite >7-fold variation in cell volume (size-scaling exponent: –0.03±0.01), supporting the hypothesis that genome size not cell size *per se* controls the size scaling of metabolic rate.

A number of mechanisms have been identified that may regulate the inter-relationships between haploid nuclear DNA content, cell volume, and cell division rates [56], [68]. Cell volume may be set by DNA content [34]. Experimental manipulations of DNA content have been shown to cause proportional changes in cell volume [69], [70], [71] and genome size has been shown to be highly correlated with cell volume across all eukaryotic organisms including algae and other unicellular eukaryotes [55]. DNA content may set the volume of the nucleus and therefore the surface area of the nuclear envelope through which nuclear control of RNA transport may control the rate of information transfer from the nucleus to the rest of the cell, regulating metabolic rate [34]. The quantity of DNA may set the minimum length of the synthesis phase (S), which may act as an important constraint on the minimum time it takes to complete the cell division cycle [72], [73]. In addition to DNA content a number of molecular mechanisms may alter the length of the synthesis phase including the density and regulation of replication origins, ploidy-dependent expression, and transient pairing interactions between homologous chromosomes [69], [74]. These mechanisms may account for similar S phase length in allohexaploid *Triticale* relative to its triploid or diploid ancestors [72], [75], and similar exponential growth rate in triploid and tetraploid cultures of *Saccharomyces cerevisiae* [69]. It is noteworthy that increases in division rates of haploid relative to tetraploid *Saccharomyces cerevisiae* is consistent with a cell volume size-scaling exponent of -0.24 (assuming a linear relationship between DNA content and cell volume), similar to the scaling observed across the two cryptic species of *Ditylum brightwellii* with a 2-fold difference in DNA content.

For many diatom species, each round of asexual reproduction results in a decrease in cell width until recovery via sexual reproduction or vegetative enlargement, resulting in ∼2 to >100-fold variation in cell volume over the life cycle with no associated change in haploid DNA content [47], [53], [57]. *Ditylum brightwellii* varies in diameter (width in girdle view) from ∼5 to slightly more than 110 µm; most of this variation occurs within species [52], [57]. The clones of *Ditylum brightwellii* used in this study were in the intermediate size range for the species (Table 1). The lack of size scaling of growth rate within species despite significant changes in cell volume found here has been observed in a large range of other diatom species over their intermediate size range [57], [76], [77], [78]. Decreases in growth rates within diatom species have been reported at both the extreme large and small cell size, and is attributed to the mechanics and costs associated with sexual reproduction [57], [76], [79]. An approximately constant growth rate within species despite significant increases in cell volume and mass requires a compensating increase in energy acquisition and reductant, with consequences for photophysiological traits across and within the cryptic species with cell volume. Nutrient acquisition rates and nutrient quotas might likely be affected as well, but were not studied in these experiments.

Light acquisition by a phytoplankton cell is a function of the light field, pigment composition, intracellular pigment concentration and arrangement and cell volume [44], [45]. Due to biophysical constraints, a larger cell will absorb fewer photons per unit pigment than a smaller cell with the same intracellular pigment concentration due to self-shading of pigment within the larger cell volume [44], [45]. Similarly a cell of the same cell volume with higher intracellular pigment concentration will absorb fewer photons per unit pigment than a physiologically equivalent cell with lower intracellular pigment concentration [44], [45]. As a consequence, under similar environmental conditions, larger species generally maintain lower intracellular pigment concentrations to maintain similar absorption cross-sections per unit pigment and tend to have lower growth efficiency (α_µ_) at low irradiance [7], [8], [36]. Consistent with these previously observed cross-species patterns, P2 has fewer PSII per unit protein, measured as PsbA, than P1, and therefore is able to maintain a similar functional absorption cross-section for PSII (compare σ_PSII_ in Table 1). In contrast, within both P1 and P2, the larger clones have more PSII per unit protein and as a result have lower functional absorption cross-sections for PSII. From the perspective of an optimized energy budget this is surprising, because smaller cells should be able to obtain relatively higher growth rates based on their smaller cell volume and ability to maintain higher intracellular pigment concentrations and high pigment-specific absorption cross-sections. The increased PSII per unit protein in the larger versus the smaller clones is instead consistent with the larger energy requirement required for maintaining the same cell division rate as the smaller clones, and the hypothesis that maximum growth rate is set by DNA content and nuclear volume and not by the biophysical constraints of the metabolic network or light acquisition capacity. The higher PSII per unit protein may also be the cause of the high susceptibility of P2B to short-term exposure to high irradiance (σ_I_, Table 1) when acclimated to low irradiance. This result differs from theoretical expectations and previous cross-species observations that found that larger species will have some additional protection from photosystem II photoinactivation compared to smaller species with the same intracellular pigment concentrations [9], [10], [46]. When grown under growth saturating irradiance there is little difference between P1S, P1B, P2S, and P2B, in repair rate capacity for PSII or susceptibility of PSII to photoinactivation with transient high light exposure (Table 1), indicating that factors other than cell size may contribute to variability in these parameters under high light stress [80].

Increases in DNA content and associated changes in cell volume have the capacity to alter whole organism traits, and could facilitate sympatric speciation [56]. Some diatom species appear to be susceptible to changes in ploidy, and this may be one of the mechanisms facilitating the high diversity of this group [53]. The increase in genetic material and increase in cell volume should favor selection for specific photophysiological traits. Genetically distinct isolates of *D. brightwellii* from different geographic regions have been shown to differ in their growth rates for a range of irradiances [52]. For example, 8 genetically different isolates (based on microsatellite data) of *D. brightwellii* from Hood Canal, Puget Sound, Washington, exhibited differences in growth rate in response to 33, 66 and 166 µmol photons m^−2^s^−1^ [81], and had lower growth rates, regardless of irradiance, relative to isolates from the Strait of Juan de Fuca at Admiralty Inlet [82]. In this study the species with the larger genome (P2) exhibits lower maximum growth rates (µ_max_) than that the species with the smaller genome (P1), regardless of significant intra-species differences in cell size (Table 2). In addition, growth efficiency (α_µ_), and induced PSII repair in response to transient high light exposure (R_PSII_), and the functional absorption cross-section for PSII (σ_PSII_) are all higher in P1 relative to P2, although not all these parameters are significantly different from one another (Table 1). The higher PSII repair and growth rates in P1 relative to P2 are consistent with the variation in these parameters with cell size across diatom species reported in previous work [9]. In addition to the variable functional responses to irradiance, the two cryptic species differ in PSII (PsbA and PsbD) content; the faster growing P1 has higher PsbA per µg protein than the slower growing P2 (Table 1), and higher ratios of PsbA:PsbD that may provide buffering capacity within the PSII repair cycle to deal with fluctuating light or excess photons under high irradiance.

Differences in photophysiology between these two clones of *Ditylum* reinforce the hypothesis that P1 and P2 likely represent cryptic species [52] and supports the hypothesis that DNA content ultimately sets maximum growth rate, while growth rate and cell size may regulate differences in many of the photophysiological traits. Evolutionary changes in cell volume associated with changes in genome size are associated with a significant size-scaling exponent for metabolic rate. In contrast the shorter-term changes in cell size associated with asexual reproduction have a relatively minor influence on metabolic rate. The size scaling of growth rate across the nascent species of *Ditylum brightwellii* is consistent with values calculated across many different species of diatoms [8], [17], [19]. Why significant changes in cell size within species result in little change in metabolic rate remains unknown and further studies into the molecular mechanisms and evolutionary origins are required. The role of nuclear and cytoplasmic volume, transport factors and cell division cycle regulation, RNA and ribosomal content, and elemental composition and ratios such as carbon-to-nitrogen-to-phosphorus in controlling metabolic rate may be promising avenues for future research [34], [56], [83], [84], [85]. Biophysical constraints on light absorption and nutrient uptake would be expected to reduce metabolic rate in larger diatoms if there is no reduction in cellular energy, and nutrient requirements (for example: cellular carbon, nitrogen and phosphorus) [42], [43]. If larger cells within species do have lower energy and nutrient requirements it is still unclear why the smaller cells were unable to similarly reduce their cellular nutrient requirements and therefore further increase their growth rates.
